# Pelvic actinomycosis: diagnostic challenges and management strategies based on a retrospective case series and literature review

**DOI:** 10.1007/s00404-026-08441-z

**Published:** 2026-06-13

**Authors:** Shuxu Tian, Chenchen Geng, Yue Wu, Kexin Huang, Shumin Chen, Xiucheng Fan, Weiwen Fan

**Affiliations:** 1 Department of Gynecology, Qingdao Women and Children’s Hospital, Qingdao University, No.217 Liaoyang Road, Qingdao, 266034 Shandong China; 2 Department of Ultrasound, Qilu Hospital of Shandong University (Qingdao), Shandong University, No.758 Hefei Road, Qingdao, 266035 Shandong China; 3 School of Medicine, Sun Yat-Sen University, No.66 Gongchang Road, Shenzhen, 518107 Guangdong China; 4 Department of Obstetrics and Gynecology, the Seventh Affiliated Hospital, Sun Yat-Sen University, No.628 Zhenyuan Road, Shenzhen, 518107 Guangdong China; 5 School of Medicine, The First Affiliated Hospital, Army Medical University, Shapingba District, No.29, Gaotanyan Street, Chongqing, 400038 China

**Keywords:** Pelvic actinomycosis, Abscess, Intrauterine device, Diagnosis, Antibiotic therapy

## Abstract

**Background:**

Pelvic actinomycosis is a rare, chronic infection caused by *Actinomyces* species, most associated with prolonged intrauterine device (IUD) use. Due to its nonspecific clinical presentation, it is frequently misdiagnosed as malignancy or other inflammatory conditions. This study aims to analyze the clinical features, diagnostic approaches, and outcomes of pelvic actinomycosis through a retrospective review of four cases and a supporting literature review.

**Case description:**

We reviewed the medical records of four female patients diagnosed with pelvic actinomycosis between 2021 and 2025. Diagnostic modalities included imaging, physical examination, cervical cytology, endometrial biopsy, and histopathological analysis. Empirical antibiotic therapy was administered to confirmed cases. All patients had a history of prolonged IUD use. Imaging frequently revealed pelvic masses with inflammatory features. In all cases, *Actinomyces* was identified via cytology or histopathology. Three of the four patients were diagnosed preoperatively, thus preventing unnecessary surgical interventions. Long-term antibiotic therapy led to clinical resolution in most patients.

**Conclusion:**

Pelvic actinomycosis should be considered in women with chronic pelvic symptoms and a history of long-term IUD use. Early identification through cytology or biopsy can reduce surgical morbidity. Increased awareness and its inclusion in the gynecologic differential diagnosis may improve patient prognoses.

## Introduction

Pelvic actinomycosis is a rare, chronic infection caused by *Actinomyces* species, a group of facultative anaerobic bacteria that more commonly cause cervicofacial and gastrointestinal infections [1]. The incidence of pelvic actinomycosis is low, and its presentation is typically insidious and nonspecific, often misdiagnosed as ovarian malignancy or pelvic tuberculosis [2]. The condition is most frequently observed in women with a prolonged history of intrauterine device (IUD) use, where it manifests as suppurative or granulomatous inflammation within the pelvic and abdominal cavities. Due to the vague nature of the symptoms, early diagnosis is often delayed, resulting in unnecessary surgical interventions.

This retrospective study analyzes four cases of pelvic actinomycosis diagnosed at the Qingdao Women and Children’s Hospital between 2021 and 2025. Of particular note, three of the four cases were diagnosed preoperatively, which helped avoid unnecessary surgical procedures and improved patient outcomes.

## Case presentation

### Case 1

**Fig. 1:**
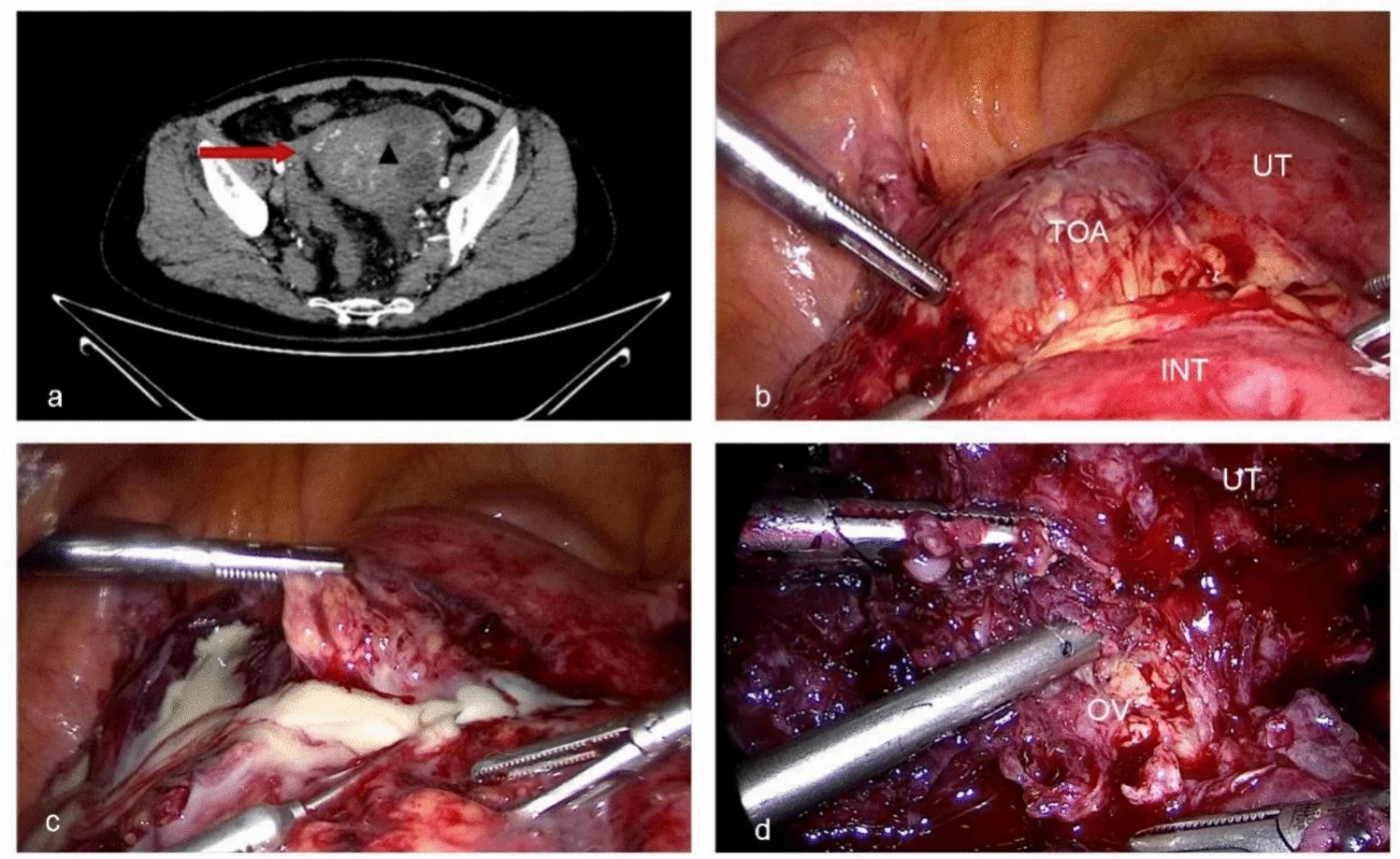
CT image and intraoperative laparoscopic findings for Case 1. **a** the CT scan demonstrates a slightly hypodense lesion in the left adnexal region, measuring 78.9 × 19.5 × 32.1 mm (indicated by the red arrow), with no enhancement after contrast administration. **b–d**. Laparoscopic images show the presence of a left tubo-ovarian abscess with dense adhesions to adjacent organs. Purulent fluid was observed draining intraoperatively. The ovary had lost its normal morphology and was severely affected by purulent infection. UT uterus; TOA tubo-ovarian abscess; INT intestine; OV ovary

A 50-year-old woman (G1P1) was admitted with a 3-week history of intermittent left lower abdominal pain accompanied by a sensation of rectal pressure. She reported poor appetite and an unintentional weight loss of 2.5 kg over the preceding month. She had a 25-year history of IUD use. Pelvic ultrasonography revealed an IUD and a hypoechoic mass measuring approximately 80 × 60 mm in the left adnexal region, characterized by a serpiginous, tubular, fluid-filled structure up to 23 mm in width with poor sound transmission. Gynecological examination identified a firm, poorly mobile, cystic-solid mass approximately 8 cm in diameter in the left adnexa, with significant tenderness and dense adhesions to the pelvic wall. Pelvic CT showed a slightly hypodense lesion in the left adnexal area measuring 78.9 × 19.5 × 32.1 mm, with no enhancement following contrast administration, and inflammatory exudative changes within the pelvis. Laboratory findings revealed leukocytosis (WBC: 13.51 × 10⁹/L), neutrophilia (83.3%), and elevated C-reactive protein (62.58 mg/L), while CA125 and HE-4 were within normal limits. Laparoscopic exploration was performed, revealing a tubo-ovarian abscess (TOA) on the left side with dense adhesions to the surrounding bowel, posterior uterine wall, and pelvic sidewall (Fig. 1). The operation was technically challenging because of dense inflammatory adhesions involving the bowel, posterior uterine wall, and pelvic sidewall; therefore, left adnexectomy was performed, and the IUD was removed. Perioperatively, the patient received intravenous cefoperazone-sulbactam (2 g IV q12h) for 1 week. Histopathological analysis of the resected tubo-ovarian mass identified *Actinomyces*, and cervical cytology revealed bacteria morphologically consistent with the organism. The diagnosis of pelvic actinomycosis with abscess formation was established. The patient recovered uneventfully and was discharged on a 3-month course of oral amoxicillin-clavulanate (875/125 mg PO q12h). She remained symptom-free during outpatient follow-up.

### Case 2

**Fig. 2:**
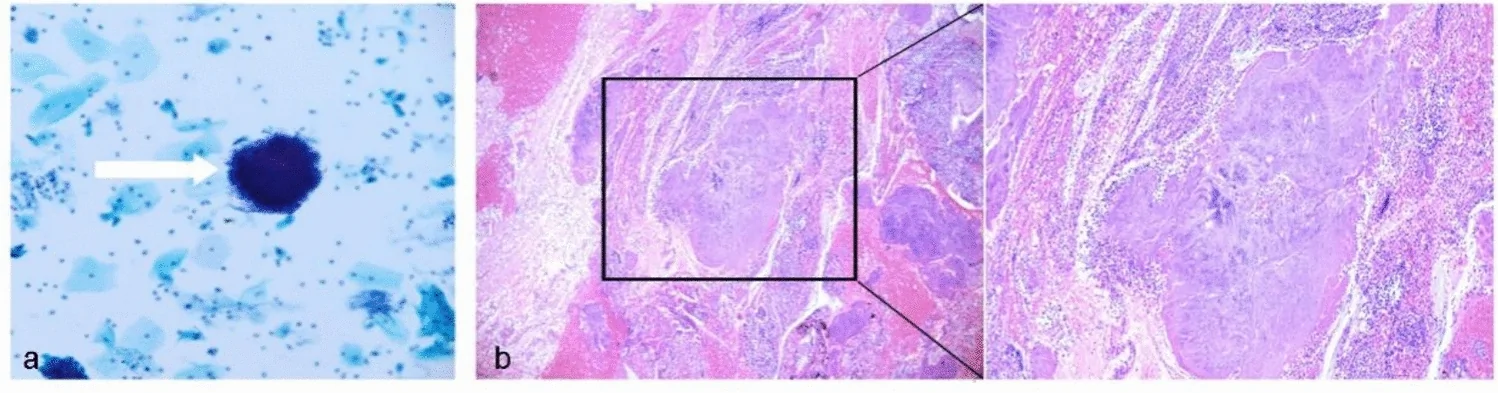
Actinomyces colonies in cervical cytology and endometrial biopsy specimens. **a** Against an inflammatory background, purple-blue mycelia arranged in a radial pattern were observed (Papanicolaou stain, × 100). **b** Clusters of purple-blue Actinomyces with surrounding inflammatory cells were identified in the endometrial tissue (hematoxylin and eosin stain: left, × 50; right, × 100)

A 49-year-old woman (G3P2) was admitted with a 4-month history of lower abdominal pain and difficulty in defecation, which had worsened over the past 3 weeks. The pain, primarily localized to the left lower abdomen, was accompanied by constipation, with post-defecation relief. Her appetite had decreased, and she experienced an 8 kg loss in the past 4 months. 3 weeks before admission, the pain intensified and was accompanied by fever, with a maximum temperature of 38.2 °C. Colonoscopy revealed a localized fistula in the lower segment of the anterior rectal wall, from which a small amount of yellowish-white purulent discharge was observed. Histopathological examination of the fistula tissue showed chronic active inflammation with ulceration. She had a history of hypertension and had used an IUD for contraception for 22 years. Laboratory findings included marked leukocytosis (WBC: 31.00 × 10⁹/L), neutrophilia (92.0%), thrombocytosis (392 × 10⁹/L), and elevated C-reactive protein (CRP: 284.84 mg/L). Serum CA-125 levels were 64.75 U/mL, while HE-4 remained within normal limits. Pelvic CT demonstrated multiple cystic low-density lesions in the bilateral adnexal regions with ill-defined and irregular borders, measuring approximately 86 × 56 × 103 mm on the left, 54 × 61 × 62 mm on the right, and 50 × 51 × 71 mm on the right lower side. The lesions exhibited mild enhancement of the septa and walls after contrast administration, but no significant enhancement within the cystic spaces. The lesions extended to involve the rectum and part of the sigmoid colon. The pelvic examination revealed a large, firm, immobile mass firmly adherent to the pelvic wall, with no clear delineation of the uterine contours, consistent with a “frozen pelvis.” A clinical diagnosis of pelvic actinomycosis with abscess formation and rectal fistula was suspected. Subsequently, cervical cytology and endometrial biopsy were performed during IUD removal, both of which confirmed the presence of *Actinomyces* species (Fig. 2) and were consistent with the diagnosis. Empiric antibiotic therapy with intravenous clindamycin (600–600 mg IV q8h) combined with oral amoxicillin (500 mg PO q8h) was initiated. After 1 week of treatment, the patient’s fever subsided, and her abdominal pain decreased. Blood tests showed normalization of the WBC count and CRP levels. After defervescence and improvement in abdominal pain, we attempted to maintain inpatient therapy. However, the patient discontinued hospitalization due to personal reasons. She was prescribed a course of oral amoxicillin/clavulanate (875/125 mg PO q12h) combined with doxycycline (100 mg PO q12h) for 3 months. The patient was lost to follow-up despite repeated attempts to contact.

### Case 3

A 51-year-old woman (G4P2) described a 4-month history of recurrent lower abdominal discomfort and bloating. The pain was relieved after flatus. She had a poor appetite and experienced significant weight loss of approximately 13 kg over the past 6 months. She had used an IUD for contraception for 15 years. Pelvic–abdominal CT showed right hydronephrosis and mid and upper hydroureter, with unclear delineation of the right ureter as it crossed the iliac vessels. The surrounding structures were disorganized, with some bowel loops and the right ovary adhered to one another. The appendix could not be clearly visualized, and signs of pelvic peritonitis and slight enlargement of the lymph nodes in the right iliac region were discovered. MRI revealed widespread abnormal signals in the right lower abdomen and pelvis, suggestive of inflammatory lesions. A right adnexal abscess was suspected. The right ureter was compressed and narrowed at the pelvic segment, causing proximal ureteral dilation. Blood work showed normal WBC and CA125 levels, while HE-4 was elevated to > 1500 pmol/L. Pelvic examination revealed a firm, fixed, and immobile mass in the right anterior pelvic region, approximately 15 × 15 cm in size, with severe adhesions to the surrounding tissues. Tenderness was noted on palpation, but there was no rebound tenderness or guarding. Given the clinical findings, pelvic inflammatory disease secondary to *Actinomyces* or tuberculosis was suspected. The IUD was removed, and cervical cytology and endometrial biopsy were performed. Both tests revealed bacterial infection, with morphology consistent with *Actinomyces*. A diagnosis of pelvic actinomycosis was confirmed, and the patient was treated with intravenous penicillin G (20 million units daily) for 2 weeks, along with the placement of a right ureteral stent to relieve obstruction. Upon discharge, she was prescribed a course of oral amoxicillin/clavulanate (875/125 mg PO q12h) for continued treatment. After 1 month, a follow-up examination revealed a reduction in the pelvic mass size to approximately 6 cm. The stent was removed after 3 months, and after another 2 months, a final follow-up revealed complete resolution of the pelvic mass, with no recurrence on imaging.

### Case 4

A 43-year-old woman (G1P1) was admitted with a 1-week history of persistent lower abdominal pain, that progressively worsened and was accompanied by fever, with a maximum temperature of 39 °C. She had experienced poor appetite and a weight loss of 2 kg over the past month. She had used an IUD for contraception for 18 years. Cervical cytology performed in June 2021 indicated atypical squamous cells of undetermined significance (ASCUS), and *Actinomyces* was observed in the smear, but no further treatment was initiated. Ultrasound revealed a right ovarian mass measuring approximately 89 × 64 × 55 mm, with heterogeneous echotexture and a hypoechoic area approximately 83 × 31 mm in size. Doppler studies indicated blood flow within the mass, and multiple smaller cystic areas were noted. CT confirmed the presence of the IUD within the uterine cavity and a markedly enlarged right adnexal mass. Blood tests revealed leukocytosis (WBC: 13.51 × 10⁹/L), neutrophilia (89.4%), thrombocytosis (440 × 10⁹/L), and elevated CRP (209.83 mg/L). Tumor markers (CA125, HE-4) were within normal limits. Pelvic examination revealed a fixed, immobile mass approximately 10 cm in diameter in the right adnexa, which was adherent to the surrounding tissues with significant tenderness but no rebound tenderness or muscle rigidity. Repeat cervical cytology showed persistent evidence of *Actinomyces*. The patient was diagnosed with pelvic actinomycosis, and empiric therapy with piperacillin-tazobactam (4.5 g IV q8h) was initiated. After 12 days of treatment, her fever subsided, and abdominal pain resolved. Upon discharge, she was continued on oral amoxicillin-clavulanate (875/125 mg PO q12h) for 3 months. Follow-up imaging confirmed the resolution of the pelvic mass, and the patient was clinically recovered.

## Literature review

Pelvic actinomycosis is an uncommon chronic bacterial infection of the pelvis caused by *Actinomyces* species. It typically manifests as localized abscesses, draining fistulas, and significant fibrosis. *Actinomyces* are opportunistic bacteria commonly residing in the oropharynx, lower esophagus, and genitourinary tract [1]. Breach of the mucosal barrier—often due to surgery, trauma, or the introduction of foreign bodies—can result in deep-seated infections. Such infections are more common in immunocompromised individuals and patients with diabetes, primarily affecting regions such as head, neck, and gastrointestinal tract, whereas pelvic involvement remains rare [2, 3]. Confirmation of diagnosis usually relies on culture or histopathological examination; however, the sensitivity is under 50% even though radially arranged hyphae are characteristic [4]. Within affected tissues, *Actinomyces* frequently form sulfur granules, which are firm, range from 100 to 1000 µm, and can often be detected visually or with low-power microscopy, aiding diagnosis [5]. Culturing under anaerobic conditions enhances specificity, but culture results may take 2 to 3 weeks, and delays in postoperative confirmation are not unusual. Additionally, exposure of samples to air can yield false negatives. Advanced techniques like 16S rRNA gene sequencing achieve perfect specificity but remain costly, limiting their routine use [6].

Emerging evidence indicates that *Actinomyces* can inhabit the female reproductive tract. An analysis of over 20,000 cervical smears found *Actinomyces*-like organisms in 0.26% of cases, though only about half were verified by culture [7]. Reports indicated that the occurrence of pelvic actinomycosis in women was closely related to the use of intrauterine devices [8, 9]. Reviewed cases consistently involved prolonged IUD retention without adequate follow-up. It is proposed that chronic irritation and local trauma from IUDs disrupt the endometrial surface and vascular supply, altering the uterine microbial ecosystem and promoting bacterial colonization and spread. Cervical cytology shows *Actinomyces*-like bacteria in up to 7% of IUD users, markedly higher than in women without IUDs, indicating increased risk [8]. Nonetheless, a positive smear does not necessarily signify an active infection. Secondary pelvic actinomycosis can also develop following intestinal infections or after pelvic surgery where hemostatic agents are used [10, 11].

The disease often presents with vague and insidious signs, frequently leading to confusion with other granulomatous or neoplastic conditions, such as ovarian or colorectal malignancies and tuberculosis, thereby complicating timely diagnosis. Early manifestations may include persistent pelvic discomfort, unintended weight loss, unusual vaginal discharge, or constipation. Physical examination sometimes reveals a poorly defined, immobile pelvic mass [3, 12]. If the urinary tract or intestines become involved, complications like ureteral blockage or bowel leakage may occur. Some patients develop acute signs such as fever and elevated inflammatory markers, including granulocytes, ESR, and CA125 levels. Imaging usually shows mass-like lesions in the abdomen or pelvis [13]. Computed tomography may reveal indistinct margins and inflammatory infiltration around the lesion, and palpation may detect a larger mass than seen on imaging. Notably, PET–CT can show increased metabolic activity mimicking malignancy [14]. Due to these nonspecific features, preoperative misdiagnosis is common, and some patients undergo extensive resection for presumed cancer [12, 14, 15].

Currently, no definitive guidelines exist for ideal treatment protocols. Extended antibiotic therapy, usually penicillin for 3 to 6 months, is widely recommended. Other agents, such as doxycycline, erythromycin, sulfonamides, streptomycin, chloramphenicol, vancomycin, clindamycin, and lincomycin, are alternatives [3]. Long-term therapy helps prevent recurrence and is generally continued for at least 1 to 2 months after symptoms are resolved. Shorter courses may suffice for cases identified early with limited lesions [16]. The necessity for surgery depends on disease severity and location. Patients with mild infection, stable vital signs, and no abscess or fistula usually only require antibiotic therapy. Surgery is reserved for refractory cases, abscesses complicated by fistulas, or life-threatening conditions.

## Case discussion

Early diagnosis of pelvic actinomycosis remains a clinical challenge due to its nonspecific presentation, which frequently mimics malignancy or other inflammatory conditions. A delayed or missed diagnosis can lead to extensive surgical interventions, complicated by the dense pelvic adhesions characteristic of the disease, which increase the risk of iatrogenic injury. Therefore, achieving a preoperative diagnosis through minimally invasive methods is critical for optimizing patient outcomes. To contextualize our findings and provide a comparative analysis, we conducted a literature search of PubMed for English-language case series of pelvic actinomycosis published between January 2000 and May 2025. Key characteristics from selected representative series were summarized for comparison with ours (Table 1). Our case series is distinguished from many prior reports by its high rate of preoperative diagnosis, which was achieved in 75% of cases (3 of 4). By utilizing cervical cytology and endometrial biopsy in conjunction with imaging, we obviated the need for extensive exploratory surgery in these patients. This finding underscores the importance of maintaining a high index of suspicion for pelvic actinomycosis in women with a pelvic mass and a history of long-term IUD use.

**Table 1:** Selected published reports of pelvic actinomycosis

Author (publication year)	Article type	Patient's symptoms	IUD	Laboratory	Key imaging features	Method of confirmation	Timing of diagnosis	Antibiotic therapy
García-García A. et al. (2017)[9]	Systematic review (*N* = 63)	Nonspecific symptoms, e.g., weight loss, abdominal pain, vaginal bleeding, and rare occasions, fever	Frequent	Elevated WBC neutrophilia, and elevated CRP commonly; tumor markers may show mild elevations in CA-125 and CA 15–3	Most commonly present as pelvic tumor-like mass	The majority by surgery, others by endometrial biopsy	The majority postoperative	Yes
Saramago, S.M. et al. (2019)[17]	Case report (*N* = 1)	Nonspecific symptoms	Yes	WBC and CRP normal	Pelvic mass, hydronephrosis and iliac lymph node enlargement	Surgery	Postoperative	Yes
Ferjaoui, M.A. et al. (2021) [18]	Retrospective study (*N* = 12)	Abdominal pain 66%, fever 33%, vaginal bleeding 25%	75%	Elevated WBC 58%, elevated CRP 58%, anemia 83%, elevated CA-125 66%	8 cases of pelvic tumor, 4 cases of intrauterine heterogeneous proliferation	75% by surgery, others by endometrial biopsy	75% postoperative, others preoperative	Yes
Koteeswaran, R. (2022) [19]	Case report (*N* = 1)	Abdominal pain, lack of appetite	Yes	Elevated WBC, CRP, CA-125	Bilateral adnexal masses	Surgery	Postoperative	Yes
Cao Y, et al. (2023)[20]	Case report (*N* = 3)	Abdominal pain, abdominal mass	Yes	WBC, CRP normal, 2/3 elevated CA-125	Ovarian space-occupying lesions involving the surrounding tissue	Surgery	Postoperative	yes
Ayoub F, et al.(2024) [21]	Case report (*N* = 2)	Lower abdominal pain	Yes	Elevated WBC, CRP	Complex pelvic mass complicated appendicitis, pelvic inflammatory disease	Surgery	Postoperative	Yes
Restaino S, et al. (2025) [22]	Case report (*N* = 2)	Abdominal pain, distention, vaginal bleeding	Yes	Elevated WBC, CRP	Pelvic mass of adnexal origin and pelvic lymphadenopathy	Surgery	Postoperative	Yes
Our study cohort	Case report (*N* = 4)	Abdominal pain, weight loss, 1/4 fever	Yes	3/4 elevated WBC neutrophilia and elevated CRP; 1/4 elevated HE-4	Pelvic tumor-like mass	1/4 by surgery, others by endometrial biopsy/cervical cytology	1/4 postoperative, others preoperative	Yes

*N* number of patients, *IUD* intrauterine device, *WBC* white blood cell, *CRP* C-reactive protein, *CA*-125 carbohydrate Antigen 125, *CA* 15–3 carbohydrate antigen 153

In all four cases, the contraceptive device was a copper-containing IUD (ring-shaped or uterine-shaped type). None of the patients had the device replaced after insertion, and all had prolonged continuous use. Based on these findings, we recommend that women using an IUD should undergo removal or replacement according to the product instructions, which are generally within 5–10 years depending on the specific device.

Furthermore, our series highlights two diagnostic nuances that warrant emphasis. First is the limited specificity of cervical cytology alone; an incidental finding of *Actinomyces*-like organisms on a Pap smear in an asymptomatic IUD user is not diagnostic of invasive disease and does not warrant treatment. Histopathologic or microbiologic confirmation is necessary. Second, severe inflammation from actinomycosis can significantly elevate tumor markers, such as the markedly high HE4 level seen in Case 3, further confounding the clinical manifestations and reinforcing the mimicry of malignancy.

## Conclusion

Pelvic actinomycosis is a rare but potentially severe infection associated with long-term IUD use, presenting with clinical manifestations that often resemble more common gynecological disorders. Early diagnosis, supported by cervical cytology or biopsy, is essential for appropriate management and avoidance of unnecessary surgery. Awareness of pelvic actinomycosis should be heightened in women with unexplained pelvic masses, especially those with a history of prolonged IUD use. This case series highlights the importance of early diagnosis and empirical antibiotic therapy in improving patient outcomes.

## Data Availability

Data are available on request from the authors due to patient confidentiality/privacy.
